# Lethal Consequences of Overcoming Metabolic Restrictions Imposed on a Cooperative Bacterial Population

**DOI:** 10.1128/mBio.00042-17

**Published:** 2017-02-28

**Authors:** Eunhye Goo, Yongsung Kang, Jae Yun Lim, Hyeonheui Ham, Ingyu Hwang

**Affiliations:** Department of Agricultural Biotechnology, Seoul National University, Seoul, Republic of Korea; University of California, Berkeley

## Abstract

Quorum sensing (QS) controls cooperative activities in many *Proteobacteria*. In some species, QS-dependent specific metabolism contributes to the stability of the cooperation. However, the mechanism by which QS and metabolic networks have coevolved to support stable public good cooperation and maintenance of the cooperative group remains unknown. Here we explored the underlying mechanisms of QS-controlled central metabolism in the evolutionary aspects of cooperation. In *Burkholderia glumae*, the QS-dependent glyoxylate cycle plays an important role in cooperativity. A bifunctional QS-dependent transcriptional regulator, QsmR, rewired central metabolism to utilize the glyoxylate cycle rather than the tricarboxylic acid cycle. Defects in the glyoxylate cycle caused metabolic imbalance and triggered high expression of the stress-responsive chaperonin GroEL. High-level expression of GroEL in glyoxylate cycle mutants interfered with the biosynthesis of a public resource, oxalate, by physically interrupting the oxalate biosynthetic enzyme ObcA. Under such destabilized cooperativity conditions, spontaneous mutations in the *qsmR* gene in glyoxylate cycle mutants occurred to relieve metabolic stresses, but these mutants lost QsmR-mediated pleiotropy. Overcoming the metabolic restrictions imposed on the population of cooperators among glyoxylate cycle mutants resulted in the occurrence and selection of spontaneous *qsmR* mutants despite the loss of other important functions. These results provide insight into how QS bacteria have evolved to maintain stable cooperation via QS-mediated metabolic coordination.

## IMPORTANCE

We address how quorum sensing (QS) has coevolved with metabolic networks to maintain bacterial sociality. We found that QS-mediated metabolic rewiring is critical for sustainable bacterial cooperation in *Burkholderia glumae*. The loss of the glyoxylate cycle triggered the expression of the stress-responsive molecular chaperonin GroEL. Excessive biosynthesis of GroEL physically hampered biosynthesis of a public good, oxalate. This is one good example of how molecular chaperones play critical roles in bacterial cooperation. In addition, we showed that metabolic restrictions in the glyoxylate cycle acted as a selection pressure on metabolic networks; there were spontaneous mutations in the *qsmR* gene to relieve such stresses. However, the presence of spontaneous *qsmR* mutants had tragic consequences for a cooperative population of *B. glumae* due to failure of *qsmR*-dependent activation of public good biosynthesis. These results provide a good example of a bacterial strategy for robust cooperation via QS-mediated metabolic rewiring.

## INTRODUCTION

The recognition of bacteria as social organisms has provided population and evolutionary biological perspectives on bacterial behavior. In *Proteobacteria*, acyl-homoserine lactone (AHL)-mediated quorum sensing (QS) controls social behaviors, including swarming motility, virulence, and biofilm formation (1–3). These social behaviors require cooperation, but the evolutionary stability of cooperation is precarious, because costly cooperative strategies are vulnerable to social cheating. Several studies have demonstrated that QS-dependent metabolism stabilizes cooperativity. In *Pseudomonas aeruginosa*, the production of such public resources is positively or negatively regulated in a QS-dependent manner to minimize production costs (3–5). The integration of metabolic information with QS stabilizes public good cooperation, as cells can cooperate only when they receive the appropriate nutritional resources (6).

**FIG 1:**
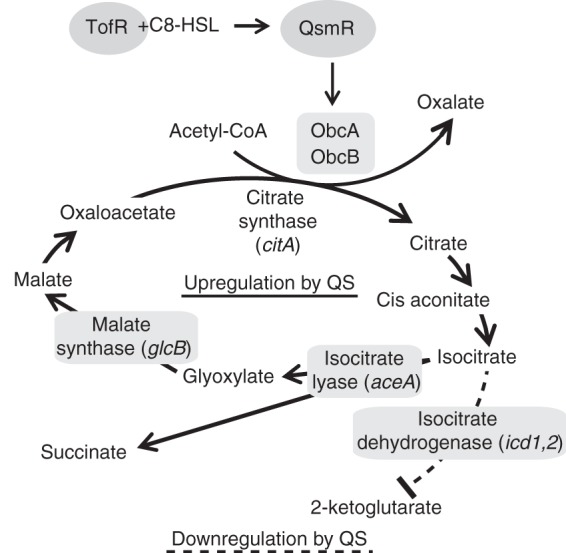
QS-regulatory metabolic diagram for *Burkholderia glumae*. When *Burkholderia* uses amino acids as a carbon source, ammonia is produced by deamination, which increases the extracellular pH (11). Among QS-dependent public goods, oxalate is biosynthesized in the branched TCA cycle to protect cells from ammonia-mediated alkaline toxicity during the stationary phase of growth (11). QsmR, the QS-dependent transcriptional regulator, directly regulates the expression of the *obcAB* genes (11). The glyoxylate cycle is composed of two specific enzymes: isocitrate lyase and malate synthase. Isocitrate dehydrogenase in the TCA cycle and isocitrate lyase both use isocitrate as a substrate. The solid and dotted lines denote activation and repression by QS, respectively.

Along with the known QS-regulatory mechanisms that support bacterial sociality, we explored whether metabolic evolution to sustain bacterial cooperativity is inherent in the structure of bacterial primary metabolic networks. To address this issue, *Burkholderia glumae* BGR1 was chosen as a model bacterium because of its dramatic QS-mediated metabolic fluctuations and metabolic plasticity (7, 8). In *B. glumae*, one LuxI-R-type QS system, TofI-R, generates *N*-octanoyl homoserine lactone (C8-HSL) as a major signaling molecule (9). The TofR and C8-HSL complex regulates expression of the *qsmR* gene, which encodes an IclR (isocitrate lyase regulator)-type transcriptional regulator (10). QsmR regulates various private and public resources, including oxalate in *B. glumae* (9–11). *Burkholderia thailandensis* E264, a nonpathogenic saprophyte, was used as a model system comparable to *B. glumae* BGR1. *B. thailandensis* contains three acyl-homoserine lactone QS circuits. The QS-1 signal (C8-HSL) is synthesized by BtaI1 complexes with BtaR1 to control aggregation, motility, and oxalate production (12). QS is essential for stationary-phase survival in both *B. glumae* and *B. thailandensis* (11). When these *Burkholderia* species use amino acids as a carbon source, deamination results in ammonia release, increasing the extracellular pH (11). Among QS-dependent public goods, oxalate is biosynthesized in the branched tricarboxylic acid (TCA) cycle to protect cells from ammonia-mediated alkaline toxicity during the stationary phase in a QsmR-dependent manner (Fig. 1) (11). The oxalate biosynthetic enzymes ObcA and ObcB use acetyl-coenzyme A (acetyl-CoA) and oxaloacetate as substrates (13, 14).

In this article, we report previously unknown functional roles of the glyoxylate cycle in bacterial cooperativity. The glyoxylate cycle allows an organism to bypass the CO_2_-generating steps in the TCA cycle (15). As a result, the glyoxylate cycle is essential for growth when acetate is the sole carbon source, as is the case for *Escherichia coli* (16). In addition to this known function of the glyoxylate cycle, we hypothesized that this cycle may play an important role in bacterial cooperativity. This hypothesis was based on previous QS-dependent transcriptome analyses of *B. glumae* that showed that the expression levels of two genes in the glyoxylate cycle, *aceA* and *glcB* (encoding isocitrate lyase and malate synthase, respectively), are QS dependent (11). We found that QsmR is bifunctional and rewires metabolic networks at the branch point of the glyoxylate and TCA cycles. Despite the presence of an intact QS system, a defective glyoxylate cycle generated metabolic stress and induced high expression of the stress-responsive chaperonin GroEL. The excessive levels of GroEL in the glyoxylate cycle mutants reduced the biosynthesis of oxalate by interrupting the oxalate biosynthetic enzyme ObcA. Metabolic imbalance in the glyoxylate cycle mutants exerted selection pressure, leading to spontaneous mutations in the *qsmR* gene. Such spontaneous mutations in the *qsmR* gene relieved the metabolic stress caused by the absence of a functional glyoxylate cycle, but they were accompanied by the loss of pleiotropy, including activation of *obcAB* gene expression, which subsequently disrupted cooperativity. From our results, we suggest that QS has evolved to be integrated into the central metabolism to maintain stable cooperation along with ensuring public good production.

## RESULTS

### Metabolic rewiring by QS at the branch point of the glyoxylate and TCA cycles.

**FIG 2:**
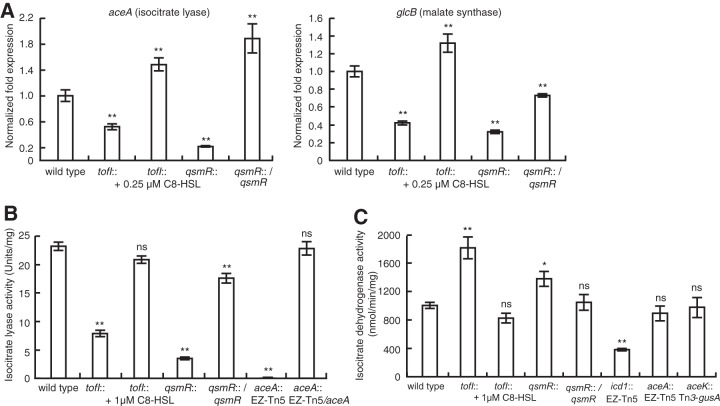
Rewiring of the metabolic networks by QsmR at the branch point of the glyoxylate and TCA cycles. (A) Expression of *aceA* and *glcB* genes, which encode isocitrate lyase and malate synthase, respectively, was activated by QsmR in *B. glumae*. (B) Isocitrate lyase activity was higher in the wild-type strain than in the QS mutants of *B. glumae*. (C) Isocitrate dehydrogenase (encoded by *icd1*) activity was higher in the *qsmR* mutant than in the wild-type strain of *B. glumae*. A mutation in the *aceK* gene, which encodes isocitrate dehydrogenase kinase/phosphatase, had no effect on isocitrate dehydrogenase activity in *B. glumae*. Error bars represent the error ranges of experiments performed in triplicate. ns, no significant difference; *, *P* < 0.05; **, *P* < 0.001.

On the basis of our previous QS-dependent transcriptome analysis, we predicted that central metabolism is QS dependent and plays an important role in *B. glumae* cooperativity. We confirmed that expression of *aceA* and *glcB* in the glyoxylate cycle is directly controlled by QsmR (Fig. 2A; see also Fig. S1A in the supplemental material). Isocitrate lyase activity was significantly higher in the wild-type strain than in the *tofI* mutant, BGS2, and the *qsmR* mutant, BGS9 (Fig. 2B). Exogenous addition of 1 μM C8-HSL to the BGS2 *tofI* mutant returned the isocitrate lyase activity to wild-type levels (Fig. 2B). Complementation strains of the *qsmR* and *aceA* mutants recovered full isocitrate lyase activity (Fig. 2B). In *B. thailandensis*, isocitrate lyase activity was also significantly higher in the wild-type E264 strain than in the *bta1* mutant, JBT101, and the *qsmR* mutant, BT09539 (Fig. S1B). However, expression of the *icd* gene, which encodes isocitrate dehydrogenase (IDH), was controlled directly by QsmR, but isocitrate dehydrogenase activity was significantly higher in the *qsmR* mutants than in the wild-type strains of *B. glumae* and *B. thailandensis* (Fig. 2C; see also Fig. S1A and C). These results indicate that QsmR rewires metabolic networks to utilize the glyoxylate cycle in preference to the TCA cycle at the branch point of these two metabolic cycles in both bacterial species.

10.1128/mBio.00042-17.1 FIG S1  QsmR regulates the branch point of the glyoxylate and TCA cycles. (A) Binding of QsmR-His to the putative promoter regions of the *aceA*, *glcB*, and *icd* genes of *B. glumae*. (B) Isocitrate lyase activity was higher in the wild-type strain than in the QS mutant of *B. thailandensis*. (C) Isocitrate dehydrogenase activity was higher in the *qsmR* mutant than in the wild-type strain of *B. thailandensis*. The *btaI1* gene encodes C8-HSL synthase in *B. thailandensis*. Error bars represent the error ranges of experiments performed in triplicate. ns, no significant difference; **, *P* < 0.001. Download FIG S1, PDF file, 0.9 MB. Copyright © 2017 Goo et al. 2017 Goo et al. This content is distributed under the terms of the Creative Commons Attribution 4.0 International license.

### Role of the QS-dependent glyoxylate cycle in *B. glumae* virulence.

**FIG 3:**
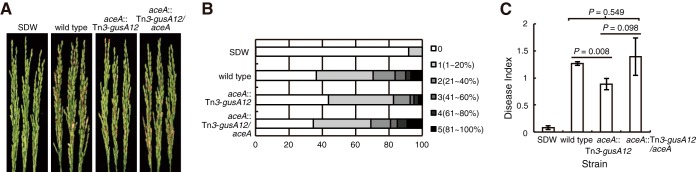
The isocitrate lyase-deficient mutant was less virulent in rice panicles than the wild-type strain. (A) The wild-type strain produced severe symptoms in the panicles, including discoloration and empty heads of grain. Panicles inoculated with the isocitrate lyase-deficient mutant displayed milder symptoms. SDW, sterile distilled water. (B and C) Disease degree (B) and index (C) were evaluated as previously described (Materials and Methods). Pathogenicity assays were repeated three times in triplicate.

Since defects in the glyoxylate cycle affected cooperativity in *B. glumae*, we examined whether the QS-dependent glyoxylate cycle plays an important role in *B. glumae* virulence. Following the inoculation of rice panicles (in the flowing stage) with the wild-type strain and the *aceA* mutant, we observed a disease index for the *aceA* mutant that was 30% lower than that for the wild-type strain (Fig. 3). Our results indicated that the glyoxylate cycle-deficient mutant was less virulent than the wild-type strain in rice panicles. Inoculation with the complementation *aceA* strain recovered the disease index to wild-type levels. These results indicate that the QS-dependent glyoxylate cycle is important for full *B. glumae* virulence in rice panicles.

### Defects in the glyoxylate cycle affect cooperative behaviors.

**FIG 4:**
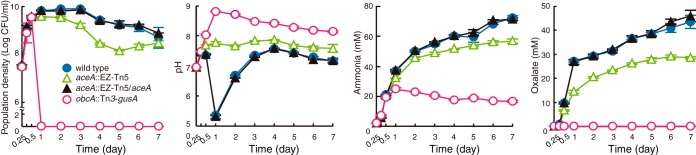
Cell viability, extracellular pH, and ammonia and oxalate production of the *aceA* mutants of *B. glumae*. Viable cell numbers of the *aceA* mutant of *B. glumae* were decreased by approximately 10% compared to that of wild-type strain, and the extracellular pH of the *aceA* mutant was maintained at 7.6 to 7.9 throughout growth, whereas the wild-type strain showed acidification and neutralization patterns. Ammonia production by the *aceA* mutant was slightly lower than that by the wild-type strain, and oxalate production by the *aceA* mutant was ~60% that of wild-type strain.

To evaluate the biological significance of such metabolic rewiring in *B. glumae* and *B. thailandensis*, we investigated whether the glyoxylate cycle plays a role in sustaining cooperativity. Growth, extracellular pH, ammonia accumulation, and oxalate production of the *aceA* mutants of *B. glumae* and *B. thailandensis* were monitored for 7 days in LB medium to identify any differences in known cooperative behaviors. Growth of the *aceA* mutants was comparable to that of the wild-type strains until the stationary phase was reached. Viable cell counts of the *aceA* mutants of *B. glumae* and *B. thailandensis* were decreased by approximately 10% compared to those of the wild-type population 1 to 3 days after subculture (Fig. 4; see also Fig. S2A). Extracellular pH of the *aceA* mutants of *B. glumae* and *B. thailandensis* remained at 7.6 to 8.2 throughout the growth period 1 day after subculture, whereas the wild-type strain exhibited slight alkalization followed by acidification and neutralization patterns (Fig. 4; see also Fig. S2A). Oxalate production by the *aceA* mutants of *B. glumae* and *B. thailandensis* was approximately 40 to 60% that of wild-type strain, which suggests a weak but continuous alkaline extracellular pH of the mutants (Fig. 4; see also Fig. S2A). These results indicate that the glyoxylate cycle plays pivotal roles in the persistence of cooperativity in *B. glumae* and *B. thailandensis* by influencing the biosynthesis of a public good, oxalate.

10.1128/mBio.00042-17.2 FIG S2  (A) Cell viability, extracellular pH, and ammonia and oxalate production by the *aceA* mutants of *B. thailandensis*. (B) Isocitrate lyase and methylisocitrate lyase exhibit concentration-dependent cross-activities in *B. glumae*. A mutation in the *prpB* gene, which encodes methylisocitrate lyase, did not affect oxalate biosynthesis, but the *aceA* and *prpB* double mutant produced less oxalate than did the *aceA* mutant. Download FIG S2, PDF file, 0.4 MB. Copyright © 2017 Goo et al. 2017 Goo et al. This content is distributed under the terms of the Creative Commons Attribution 4.0 International license.

To determine the cross-activities of isocitrate lyase and methylisocitrate lyase encoded by the *prpB* gene in *B. glumae*, levels of oxalate biosynthesis were measured in the *prpB* mutant and the *aceA* and *prpB* double mutant of *B. glumae*. The *aceA* and *prpB* double mutant produced less oxalate than the *aceA* mutant; however, no significant difference was found between the *prpB* mutant and the wild-type strain (see Fig. S2B). These results indicate that isocitrate lyase and methylisocitrate lyase have some cross-activities but that methylisocitrate lyase alone does not influence oxalate biosynthesis significantly in *B. glumae*.

### Interruption of ObcA by GroEL.

**FIG 5:**
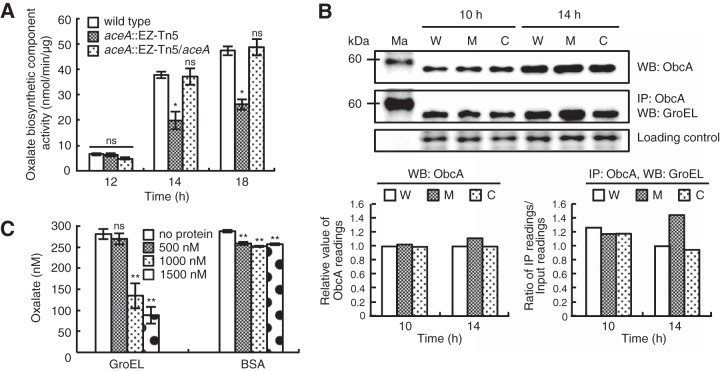
Physical interruption of the oxalate biosynthetic enzyme ObcA by metabolic stress-responsive GroEL in the *aceA* mutant of *B*. *glumae*. (A) The *aceA* mutant had approximately 50% of the oxalate biosynthetic activity of the wild-type strain. (B) Affinity pulldown experiments using anti-ObcA and anti-GroEL antibodies confirmed that GroEL interacts with ObcA, and the elevated levels of GroEL in the *aceA* mutant resulted in greater trapping of ObcA than was seen with the wild-type strain. Relative ObcA values were obtained by comparison with the wild-type values at two different time points as described previously (28). Ma, W, M, C, WB, and IP denote molecular markers, wild-type strain, *aceA* mutant, the complemented strain, Western blotting, and immunoprecipitation, respectively. The image of the SDS-PAGE gel stained with Coomassie brilliant blue R-250 (Bio-Rad) shows that samples were loaded equally in all lanes. (C) ObcA activity was decreased by approximately 4%, 52%, and 68% by addition of 500, 1,000, and 1,500 nM GroEL, respectively, compared to the control without GroEL *in vitro*. ObcA activity was not significantly affected by addition of 500, 1,000, and 1,500 nM BSA. Error bars represent the error ranges of experiments performed in triplicate. ns, no significant difference; *, *P* < 0.05; **, *P* < 0.001.

To identify the molecular mechanisms underlying the significant reduction in oxalate biosynthesis in the *B. glumae aceA* mutant, we measured the transcriptional and translational levels of *obcA* in the wild-type strain and *aceA* mutant. No difference between the wild-type strain and the *aceA* mutant was observed in the transcriptional or translational levels of *obcA* (Fig. 5B; see also Fig. S3). However, oxalate biosynthetic activity within the cells decreased by approximately 50% in the *B. glumae aceA* mutant strain compared to the wild-type strain (Fig. 5A). This was in agreement with the secreted oxalate concentration and extracellular pH. To determine the mechanisms involved in decreasing oxalate biosynthetic activity in the *aceA* mutant, we performed a transcriptome sequencing (RNAseq) comparison of the wild-type strain and the *aceA* mutant strain. We found that expression of genes encoding stress-responsive chaperones or chaperonins, such as GroEL, was approximately 30-fold higher in the *aceA* mutant than in the wild-type strain (Table 1). The expression level of *groEL* was significantly higher in the *aceA* mutant than in the wild-type strain at both the transcriptional and translational levels (see Fig. S4). These results prompted us to hypothesize that highly expressed GroEL in the *aceA* mutant interacts with ObcA and interferes with its activity. GroEL protein was pulled down using an anti-ObcA antibody in affinity pulldown experiments in both the wild-type strain and *aceA* mutant (Fig. 5B), which indicated that ObcA and GroEL interact. Interestingly, even though the same amounts of ObcA were pulled down from all samples, the amount of ObcA that interacted with GroEL was higher in the *aceA* mutant than in the wild-type strain (Fig. 5B). The higher level of GroEL in the *aceA* mutant relative to the wild-type strain was in accordance with the RNAseq transcriptomic data, which showed a higher level of GroEL in the *aceA* mutant than in the wild-type strain (see Fig. S4). To demonstrate that the interaction between GroEL and ObcA inhibits the enzymatic activity of ObcA, we performed an ObcA activity assay *in vitro*. Addition of 500, 1,000, and 1,500 nM GroEL reduced ObcA activity by approximately 4%, 52%, and 68%, respectively, compared to the control without GroEL (Fig. 5C). ObcA activity was not affected by addition of the nonspecific protein bovine serum albumin (BSA) (Fig. 5C). These results suggested that the reduced activity of ObcA in the *aceA* mutant was due to physical interruption of ObcA by excess GroEL.

10.1128/mBio.00042-17.3 FIG S3  Transcriptional and translational levels of *obcA* of the wild-type strain and the *aceA* mutant of *B. glumae* were similar. (A) The expression level of *obcA* was measured by *obcA*–*gusA* transcriptional fusion (*obcA*::Tn*3*-*gusA83*) in the wild-type strain and the *aceA* mutant of *B. glumae*. Error bars represent the error ranges of experiments performed in triplicate. (B) Immunoblot analysis using an anti-ObcA antibody showed no significant differences in the levels of ObcA among the wild-type, *aceA* mutant, and *glcB* mutant strains. We used strain *obcA*::Tn*3*-*gusA83* as a negative control. His-tagged ObcA (ObcA-His) was overexpressed in *E. coli* BL21(DE3), and total protein extracts were subjected to immunoblot analysis. M indicates molecular weight markers. Download FIG S3, PDF file, 0.7 MB. Copyright © 2017 Goo et al. 2017 Goo et al. This content is distributed under the terms of the Creative Commons Attribution 4.0 International license.

10.1128/mBio.00042-17.4 FIG S4  The expression level of groEL was significantly higher in the *aceA* mutant than in the wild-type strain. (A) Expression of the *groEL* gene was determined by qRT-PCR. (B) Immunoblot analysis using an anti-GroEL antibody. Relative GroEL expression values were estimated by comparing with the wild-type at two different time points. Ma, W, M, and C denote molecular markers, the wild-type strain, the *aceA* mutant, and the complemented strain, respectively. Error bars represent the error ranges of experiments performed in triplicate. **, *P* < 0.001. Download FIG S4, PDF file, 0.5 MB. Copyright © 2017 Goo et al. 2017 Goo et al. This content is distributed under the terms of the Creative Commons Attribution 4.0 International license.

**TABLE 1:** Expression of stress-responsive chaperone and chaperonin genes and the *citA* gene in the *aceA* mutant as analyzed by RNAseq

Gene	Locus_ID	Reads per kilobase per million mapped reads (RPKM)		
		BGR1 (wild type)	BICL39 (*aceA*::EZ-Tn*5*)	BICL39C (*aceA*::EZ-Tn*5*/*aceA*)
*groES*	bglu_1g07140	657	23,268	5,352
*groEL*	bglu_1g07150	893	26,979	8,264
*grpE*	bglu_1g06330	75	359	95
*dnaK*	bglu_1g06340	162	1,592	329
*dnaJ*	bglu_1g06350	61	419	106
*citA*	bglu_2g08280	736	3,314	853

### Metabolic stress in the *aceA* mutant.

**FIG 6:**
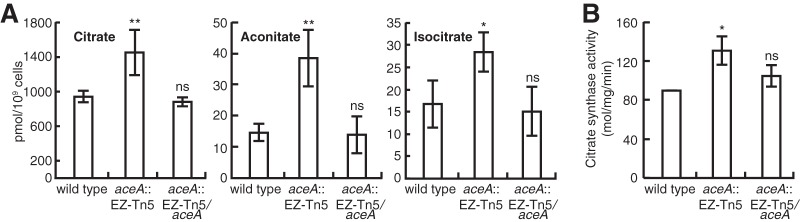
The *aceA* mutant experiences physiological stresses due to metabolic imbalance. (A) Concentrations of citrate, aconitate, and isocitrate were higher in the *aceA* mutant than in the wild-type strain. (B) Citrate synthase activity was higher in the *aceA* mutant than in the wild-type strain. Error bars represent the error ranges of experiments performed in triplicate. ns, no significant difference; *, *P* ≤ 0.1; **, *P* < 0.05.

Based on the differential expression of multiple genes encoding chaperones and chaperonins in the *aceA* mutant of *B. glumae*, we hypothesized that the mutant may experience intracellular physiological stress. Since the *aceA* mutation blocks the glyoxylate cycle, we assumed that either isocitrate dehydrogenase (IDH) activity is increased to mitigate metabolic stress in the *aceA* mutant or isocitrate, aconitate, and citrate accumulation is greater in the mutant than in the wild-type strain. IDH activity in the *aceA* mutant was not significantly different from that of the wild-type strain (Fig. 2C). However, concentrations of isocitrate, aconitate, and citrate were significantly higher in the *aceA* mutant than in the wild-type strain (Fig. 6A). Consistent with these results, both the transcriptional level of the *citA* gene, which encodes citrate synthase, and its enzymatic activity were higher in the *aceA* mutant than in the wild-type strain (Fig. 6B; Table 1). These results indicate that the *aceA* mutant experiences physiological stresses due to metabolic imbalance, which is supportive of the concept of elevated expression of stress-responsive chaperones or chaperonins, including GroEL.

### Emergence of spontaneous mutations in the *qsmR* gene in glyoxylate cycle-defective strains.

**FIG 7:**
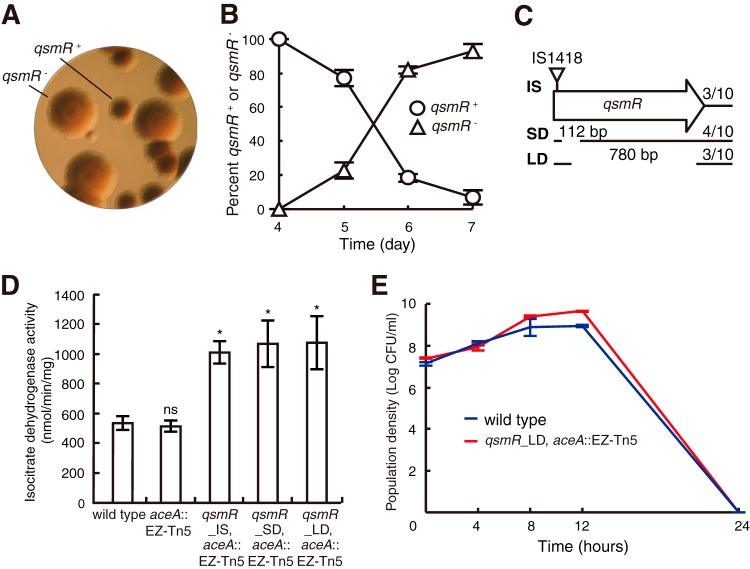
Morphological appearance of spontaneous glyoxylate cycle mutants. (A) Colonies of the spontaneous *qsmR* mutants were morphologically distinct from those of *qsmR*-positive (*qsmR*^+^) cells. Colonies were observed under a dissecting microscope at ×30 magnification. (B) The *qsmR* mutants appeared at day 4 after subculture and reached approximately 90% of the total viable population on day 7. (C) Among the 10 *qsmR* mutants characterized, 3 had an insertion of IS*1418* at the 5′ end of the gene, and 4 and 3 had 112-bp and 780-bp internal deletions, respectively. Fractions at the right end indicate the frequencies of each type of mutant among the total 10 mutants. IS, SD, and LD indicate insertion, small deletion, and large deletion, respectively. (D) Isocitrate dehydrogenase activity in three types (IS, SD, and LD) of spontaneous *qsmR* mutants was higher than in the wild-type strain or the *aceA* mutant. (E) Fitness experiments comparing the wild-type strain and the spontaneous *qsmR* mutant_LD strain. The spontaneous *qsmR* mutant LD strain did not benefit from the presence of the wild type. Error bars represent the error ranges of experiments performed in triplicate. ns, no significant difference; *, *P* < 0.05.

Such continuous intracellular metabolic imbalance may not be endurable by mutants with a nonfunctional glyoxylate cycle. Since we observed morphologically distinct colonies when glyoxylate cycle-defective mutants were cultured in the preliminary experiment, colony morphology was evaluated at various culture time points. We collected samples from batch cultures in LB broth each day (days 0, 1, 2, 3, 4, 5, 6, and 7). The samples were serially diluted, spread on an LB agar plate, and then incubated for an additional day. When colonies were observed under a dissecting microscope, no morphologically distinct mutants were observed in samples collected before day 2 of batch culture in LB broth. However, after 3 to 4 days of batch culture in LB broth, morphologically distinct mutants of *B. glumae* appeared in the *aceA* mutant cultures (Fig. 7A; see also Fig. S5). The percentage of mutant colonies in the population increased to more than 90% after 7 days (Fig. 7B; see also Fig. S5). When 7-day-old cultures of the *aceA* mutant were subcultured in LB broth after dilution to approximately 1 × 10^7^ CFU/ml and grown for 30 h, the extracellular pH reached 8.9, and no survivors were detected (see Fig. S6). To characterize such spontaneously occurring mutants, two mutants that were siblings were subjected to whole-genome resequencing. We found that IS*1418*, an insertion sequence (IS) 865 bp in length that carries 15-bp inverted repeats with a target duplication of 3 bp (17), was inserted in the *qsmR* gene in each mutant. On the basis of whole-genome resequencing data, we amplified the *qsmR* gene region of another 30 mutants. PCR products representing three different mutation types, insertion, small deletion, and large deletion, were observed (Fig. 7C). We selected three insertion mutants (*qsmR*_IS), four small-deletion mutations (*qsmR*_SD), and three large-deletion mutants (*qsmR*_LD) to determine the nature of the mutations by sequencing (Fig. 7C). These spontaneous *qsmR* mutants exhibited elevated IDH activities due to derepression of *icd* genes (Fig. 7D), which indicates that instinctive coping mechanisms to relieve metabolic stresses are operational in the glyoxylate cycle mutants. However, these mutants consequently failed to activate oxalate biosynthesis (see Fig. S7A). Thus, due to the toxic alkaline extracellular pH, the spontaneous *qsmR* mutants did not survive stationary phase after they were isolated, as previously observed in the *qsmR* mutant, BGS9 (see Fig. S7A) (11). To determine whether the spontaneous *qsmR* mutants are cheaters, we monitored the population density, pH, oxalate, and ammonia in mixed culture with the wild type. Both the wild type and the spontaneous mutants failed to survive in mixed cultures (Fig. 7E; see also Fig. S7B). This population collapse was due to the alkaline condition caused by ammonia accumulation by the wild type and the spontaneous mutants and also to the level of oxalate being insufficient to neutralize it in mixed culture (Fig. 7E; see also Fig. S7B).

10.1128/mBio.00042-17.5 FIG S5  Colony morphology in the *aceA* mutants cultured in LB broth after 4 to 6 days of growth was observed under a dissecting microscope at ×15 and ×30 magnification. Colonies of the spontaneous *qsmR* mutants increased in number gradually and reached approximately 80% of the total viable population on day 6. Download FIG S5, PDF file, 0.9 MB. Copyright © 2017 Goo et al. 2017 Goo et al. This content is distributed under the terms of the Creative Commons Attribution 4.0 International license.

10.1128/mBio.00042-17.6 FIG S6  Cell viability and extracellular pH of 7-day-old wild-type, *aceA*::EZ-Tn*5*, and *aceA*::EZ-Tn*5*/*aceA* cells after regrowth of diluted cultures for 30 h in fresh LB broth. Error bars represent the error ranges of experiments performed in triplicate. Download FIG S6, PDF file, 0.3 MB. Copyright © 2017 Goo et al. 2017 Goo et al. This content is distributed under the terms of the Creative Commons Attribution 4.0 International license.

10.1128/mBio.00042-17.7 FIG S7  (A) Growth, extracellular pH, ammonia accumulation, and oxalate production of the three types of *qsmR* mutants. All mutant phenotypes were identical to those of the *qsmR*::Ω mutant. The phenotypes of the *aceA* mutant are shown for comparison. IS, SD, and LD indicate insertion, small deletion, and large deletion, respectively. (B) Fitness experiments involving the wild-type strain and the spontaneous *qsmR* mutant_IS or SD strain. The spontaneous *qsmR* mutant_IS or SD did not benefit from oxalate biosynthesis by the wild type. Both the wild-type strain and mutants in a mixed culture died during the stationary phase due to an alkaline pH caused by an oxalate level insufficient to neutralize the accumulated ammonia. Error bars represent the error ranges of experiments performed in triplicate. Download FIG S7, PDF file, 0.4 MB. Copyright © 2017 Goo et al. 2017 Goo et al. This content is distributed under the terms of the Creative Commons Attribution 4.0 International license.

## DISCUSSION

Positive control of the glyoxylate cycle by QS allowed us to predict that the QS-dependent glyoxylate cycle may have an important role in sustaining cooperativity. In fact, it is known that the glyoxylate cycle is positively controlled by QS in *B. cepacia*, *Yersinia pestis*, and *P. aeruginosa* (18–20). Such upregulation of the glyoxylate cycle by QS provides advantages for growth on certain carbon resources (19, 20). However, the biological significance of positive control of the glyoxylate cycle by QS is unknown. In this study, we explored whether the QS-dependent glyoxylate cycle plays a role in bacterial sociality.

We first showed that QsmR rewires metabolic networks at the branch point of the glyoxylate cycle and TCA cycle in *B. glumae*. This indicated that QsmR functions as a transcriptional activator and a repressor, which is not unusual because transcriptional regulators belonging to the IclR type are often bifunctional (21). Such metabolic rewiring by bifunctional QsmR raised questions as to what the underlying reasons are and whether it is related to bacterial cooperativity. We chose *B. thailandensis* E264 as a system comparable to *B. glumae*, because it also exhibited QmsR-dependent oxalate biosynthesis (11) as well as an 80.53% identity with *B. glumae* BGR1 at the genome level, as calculated by average nucleotide identity (ANI) based on BLAST+ (ANIb) parameters (22). Concerns about the possibility that QsmR-mediated metabolic rewiring is a peculiar phenomenon in *B. glumae* were dismissed since expression of *aceA*, *glcB*, and *icd* genes in *B. thailandensis* exhibited the same gene regulation patterns as those observed in *B. glumae*. This phenomenon appears to be widely distributed among the members of the *Burkholderia* genus. Recent QS-dependent transcriptome analysis in *B. thailandensis* showed that expression of both the *aceA* and *glcB* genes is not controlled by QS (12). However, this may be due to different culture conditions. For QS-dependent transcriptome analysis, the strains of *B. thailandensis* were grown in LB supplemented with 50 mM MOPS (morpholinepropanesulfonic acid) (12) whereas expression of both the *aceA* and *glcB* gene was estimated in *B. thailandensis* cells grown in LB broth without any buffer in this study.

Since methylisocitrate lyase encoded by the *prpB* gene has cross-activity with isocitrate lyase in *B. pseudomallei* and *Mycobacterium tuberculosis* (23, 24), we expected a similar phenomenon in *B. glumae*. Cross-activity of methylisocitrate lyase and isocitrate lyase was detectable; however, the level of the cross-activity was not high enough to substitute isocitrate lyase with methylisocitrate lyase in the *aceA* mutant. Thus, the activity of methylisocitrate lyase has a negligible effect on oxalate biosynthesis in the wild-type strain.

Similarly to *E. coli*, the glyoxylate cycle in *B. glumae* is essential for growth on acetate (see Fig. S8 in the supplemental material). The glyoxylate cycle is important for the survival of pathogens inside the host and provides a physiological benefit to certain pathogenic microorganisms (19, 20, 24). A similar role of the glyoxylate cycle was observed in *B. glumae* since the *aceA* mutant was less virulent than the wild-type strain in rice panicles (Fig. 3). In addition to these biological roles of glyoxylate cycle, we were interested in the biological meaning of QS dependency in the glyoxylate cycle and in QsmR-mediated metabolic rewiring.

10.1128/mBio.00042-17.8 FIG S8  The population densities of each strain grown in M9 minimal media with 0.2% acetate were monitored. The glyoxylate cycle is required for *B. glumae* growth on acetate as the sole carbon source. Error bars represent the error ranges of experiments performed in triplicate. Download FIG S8, PDF file, 0.3 MB. Copyright © 2017 Goo et al. 2017 Goo et al. This content is distributed under the terms of the Creative Commons Attribution 4.0 International license.

It was interesting that the glyoxylate cycle affects biosynthesis of a public resource, oxalate. We then explored why and how the glyoxylate cycle affects oxalate biosynthesis in *B. glumae*. Our systematic analyses of TCA cycle intermediates and the transcriptome of glyoxylate cycle-defective mutants led us to find that blocking of the glyoxylate cycle leads to stressful conditions and triggers high-level expression of GroEL. Considering that wild-type cells have normal metabolic conditions in the presence of normal concentrations of citrate, aconitate, and isocitrate, it was clear that the *aceA* mutant experiences stressful metabolic conditions due to significantly high concentrations of these metabolites. Elevated expression of stress-responsive chaperones strongly supported the concept that physiological conditions in the glyoxylate cycle defectives are indeed stressful. The idea of interaction of the oxalate biosynthetic enzyme ObcA with GroEL was supported by the results of affinity pulldown experiments in both the wild-type strain and *aceA* mutant. In general, GroEL is a chaperonin that facilitates the proper folding of proteins, but it has been reported that an excess of certain chaperones exerts a negative effect on protein production (25). Our current results do not provide evidence that GroEL is required for full activity of ObcA. Rather, the *in vitro* ObcA activity assay suggested that excess GroEL hampered ObcA activity, resulting in reduced oxalate production (Fig. 5C). These findings indicated that the metabolic flow through the glyoxylate cycle is essential for producing oxalate as a public good in a QS-dependent manner in *B. glumae*.

In glyoxylate cycle-defective mutants, metabolic stresses were exerted as a selection pressure on the metabolic networks, resulting in the occurrence of spontaneous mutations in the *qsmR* gene to derepress expression of *icd* genes. These spontaneous mutations in the *qsmR* gene had a direct effect on the biosynthesis of public goods (see Fig. S7A), which is quite different from nonsocial adaptation, in which an individual’s fitness would increase by derepressing transcriptional repressor PsdR, for example, in *P. aeruginosa* (26). Spontaneous mutations in the *qsmR* gene were not observed in the wild-type *B. glumae* strain under the same growth conditions as those of the *aceA* mutant. Occasionally, QS enhancement of extracellular stress responses, such as the oxidative stress response, acts as a counterselective force for the appearance and survival of QS cheaters. However, intracellular metabolic stress acts as a pressure on individual cells to develop genomic mutations to relieve such stress, even though these mutations lost all of their attributes under the control of the QS-dependent QsmR. Therefore, intracellular metabolic homeostasis is an important issue in *Burkholderia* cooperative cells. It might be assumed that spontaneous *qsmR* mutants in the glyoxylate cycle-defective *B. glumae* strains are cheaters. However, these mutants are not the cheaters, because three types (IS, SD, and LD) of spontaneous *qsmR* mutant did not benefit from mixed culture with the wild-type oxalate producer (Fig. 7E; see also Fig. S7B). Mutations that inactivate *qsmR* were the fastest means of rescuing the metabolic stress but did not benefit individual cells.

In *B. glumae*, the QS-dependent glyoxylate cycle functions to support bacterial cooperativity, as well as to provide a metabolic bypass. Our results reveal important physiological roles of the glyoxylate cycle in cooperative bacteria and suggest that the glyoxylate cycle may represent a target for the development of chemical agents to control bacterial social behaviors, such as the virulence of pathogens. This may also be a good example of overcoming metabolic restrictions caused by blocking a critical metabolic flow in a population of cooperators.

## MATERIALS AND METHODS

### Bacterial strains and growth conditions.

Bacterial strains and plasmids used in this study are listed in Table S1 in the supplemental material. Strains of *B. glumae* and *B. thailandensis* were grown in Luria-Bertani (LB) broth (Affymetrix, Santa Clara, CA) (0.1% tryptone, 0.5% yeast extract, and 0.5% NaCl [all wt/vol]) at 37°C.

10.1128/mBio.00042-17.9 TABLE S1 Bacterial strains and plasmids. Download TABLE S1, DOCX file, 0.03 MB. Copyright © 2017 Goo et al. 2017 Goo et al. This content is distributed under the terms of the Creative Commons Attribution 4.0 International license.

### Quantitative reverse transcription-PCR (qRT-PCR).

Total RNAs from *B. glumae* BGR1, BGS2 (BGR1 *tofI*::Ω), BGS9 (BGR1 *qsmR*::Ω), and S9NC5 (BGR1 *qsmR*::Ω/*qsmR*), grown in LB medium at 37°C for 10 h after subculture, were extracted using RNeasy minikits (Qiagen, Venlo, Netherlands), as described by the manufacturer. Total RNA was treated with RNase-free DNase I (Ambion, Waltham, MA) to remove genomic DNA. Total RNA (1 µg) was subjected to reverse transcription into cDNA using Moloney murine leukemia virus (MMLV) reverse transcriptase (Promega, Madison, WI) and incubation for 1 h at 42°C. Primer pairs used for qRT-PCR are listed in Table S2. The 16S rRNA gene served as the positive control. Transcriptional levels were determined using SsoFast Eva Green SuperMix (Bio-Rad, Hercules, CA) and a CFX96 Real-Time PCR system (Bio-Rad). The thermal cycling parameters were as follows: 95°C for 30 s, followed by 40 cycles of 95°C for 5 s and 60°C for 5 s. All PCRs were performed in triplicate, and all data were normalized to the expression levels of the 16S rRNA gene using Bio-Rad CFX Manager software.

10.1128/mBio.00042-17.10 TABLE S2 Primers used in this study. Download TABLE S2, DOCX file, 0.02 MB. Copyright © 2017 Goo et al. 2017 Goo et al. This content is distributed under the terms of the Creative Commons Attribution 4.0 International license.

### Isocitrate lyase activity assay.

Isocitrate lyase activity was measured as described previously (27). Briefly, isocitrate was converted to succinate and glyoxylate by isocitrate lyase. During a reaction with phenylhydrazine, glyoxylate formed phenylhydrazine-glyoxylate, and the absorbance of this compound was measured at 324 nm every 60 s for 5 min. Enzymatic activity (in units per milliliter) was calculated as follows: Δ*A*_324nm_/min sample − Δ*A*_324nm_/min blank)/millimolar extinction coefficient of phenylhydrazine-glyoxylate at 324 nm.

### Isocitrate dehydrogenase activity assay.

Isocitrate dehydrogenase activity was measured using an isocitrate dehydrogenase activity assay kit (BioVision, Milpitas, CA) according to the manufacturer’s instructions. We measured the absorbance at 450 nm every 1 min in a microplate reader (PerkinElmer, Waltham, MA) and used only the data obtained within the linear range to calculate isocitrate dehydrogenase activity using the NADH standard curve, as described by the manufacturer.

### Measurement of secreted oxalate and ammonia.

Secreted oxalate and ammonia levels were measured as described previously (11).

### Overexpression and purification of GroEL.

The *groEL* gene of *B. glumae* was amplified using the primers listed in Table S2. The amplified product was cloned into NdeI and HindIII restriction sites of a pET28b expression vector (Merck, Darmstadt, Germany), resulting in pET28b-groEL (see Table S1). His-GroEL was overexpressed in *Escherichia coli* BL21(DE3), which was induced by adding 0.5 mM isopropyl β-d-thiogalactopyranoside, followed by additional growth for 14 h at 20°C. His-GroEL was purified in a buffer A, which contained 50 mM Tris-Cl (pH 8.0), 100 mM NaCl, and 5% (vol/vol) glycerol, using an immobilized metal affinity column (GE Healthcare, Chicago, IL) equilibrated with buffer A and then eluted with buffer A containing 500 mM imidazole. Purified His-GroEL was dialyzed against buffer A overnight at 4°C.

### Oxalate biosynthetic activity assay.

His-ObcA and His-Obc1* were purified, and 50 nM His-ObcA and 800 nM His-Obc1* were used for oxalate biosynthetic activity assay, as described previously (14). Oxalate biosynthetic activity and oxalate levels were measured as described previously (11, 13, 14).

### Coimmunoprecipitation (Co-IP) and Western blot analysis.

Co-IP was performed using a Pierce Direct IP kit (Thermo Fisher Scientific, Waltham, MA) according to the manufacturer’s instructions. An anti-ObcA antibody (AbFrontier, Seoul, South Korea) (2 µg) was coupled to AminoLink Plus coupling resin (Thermo Fisher Scientific). Cell lysates from *B. glumae* BGR1, BICL39 (BGR1 *aceA*::EZ-Tn*5*), and BICL39C (BGR1 *aceA*::EZ-Tn*5*/*aceA*) containing 1 mg of protein were added to the anti-ObcA-coupled resin in a spin column and mixed at 4°C for 24 h. Immunoprecipitates were eluted, followed by the addition of 5× nonreducing lane marker sample buffer. Samples were boiled for 5 min, cooled to room temperature, and separated by sodium dodecyl sulfate-polyacrylamide gel electrophoresis (SDS-PAGE). Samples were then transferred to nitrocellulose membranes. An anti-GroEL antibody (Abcam, Inc., Cambridge, United Kingdom) was used to detect GroEL protein interacting with ObcA protein. The immunoreactive bands were detected using ECL reagents (Bio-Rad) and captured by ChemiDoc XRS+ (Bio-Rad). The number of pixels per inch of each band was measured using Image Lab version 2.0.1 software (Bio-Rad). For Western blot analysis, the number of pixels measured in the wild-type strain at two different time points was set to 1, and the values for the *aceA* mutant and complemented strains were normalized to this value to allow ratio comparisons. For IP and Western blot analyses, the IP readings were divided by the input readings to obtain ratios (28).

### Quantitative analysis of citrate, aconitate, and isocitrate.

Levels of citrate, aconitate, and isocitrate in *B. glumae* BGR1, BICL39 (BGR1 *aceA*::EZ-Tn*5*), and BICL39C (BGR1 *aceA*::EZ-Tn*5*/*aceA*) were analyzed by capillary electrophoresis time of flight mass spectrometry, as described previously (7, 29, 30). All experiments were performed using at least three independent replicates.

### Citrate synthase activity assay.

Citrate synthase activity was measured using a citrate synthase assay kit (Sigma-Aldrich, St. Louis, MO, USA) and a multilabel plate reader (PerkinElmer) according to the instructions of the manufacturers.

### RNAseq analysis.

Total RNAs from *B. glumae* BGR1, BICL39 (BGR1 *aceA*::EZ-Tn*5*), and BICL39C (BGR1 *aceA*::EZ-Tn*5*/*aceA*), grown in LB medium at 37°C for 12 h after subculture, were extracted using RNeasy minikits (Qiagen) following the manufacturer’s protocols. Extracted total RNA was treated with RNase-free DNase I (Ambion) to remove DNA. The quantity and quality of the total RNA were evaluated using RNA electropherograms (Agilent 2100 Bioanalyzer, Santa Clara, CA) and by assessing the RNA integrity number (RIN). An 8-µg volume of total RNA from each sample with a RIN value greater than 8.0 was used as the starting material and was treated with a MICROBExpress mRNA enrichment kit (Invitrogen, Carlsbad, CA). The resulting mRNA samples were processed for the sequencing libraries using an Illumina mRNASeq sample preparation kit (Illumina, San Diego, CA) following the manufacturer’s protocols. One lane per sample was used for sequencing by Illumina Genome Analyzer IIx (Illumina) to generate nondirectional, single-ended, 36-bp reads. Quality-filtered reads were mapped to the *B. glumae* BGR1 genome sequence (NCBI RefSeq assembly accession no. GCF_000022645.2; https://www.ncbi.nlm.nih.gov/assembly/GCF_000022645.2/) using the BWA package (31). The mRNA reads were normalized to reads per kilobase per million (RPKM) mapped reads (32).

### Identification of the mutations in spontaneous mutant strains.

Chromosomal DNA was isolated from 10 spontaneous mutants cultured in LB broth using a method previously published by Sambrook et al. (33). To identify the mutation positions, 2 of the 10 mutants (BICL39_LY and BICL39_C) were subjected to whole-genome resequencing. Quantitation and quality assessment of the 2 samples were carried out using PicoGreen (Invitrogen) on a Victor 3 fluorometer (PerkinElmer). Illumina shotgun libraries from gDNA were prepared and sequenced on one lane of an Illumina HiSeq 2500 sequencer. Resequenced Illumina HiSeq data were trimmed using Trimmomatic software (34) and aligned to the *B. glumae* BGR1 genome using the BWA package (31). DNA variants, including single nucleotide polymorphisms, insertions, and deletions, between libraries were detected using SAMtools (35, 36) and FreeBayes (37). Subclonal single nucleotide variants were detected using the deepSNV package in R (38, 39). Gene mutation candidates were visualized and validated using Integrative Genomics Viewer (40). On the basis of whole-genome resequencing data, we amplified the *qsmR* gene region of another 30 mutants using the qsmR-F and qsmR-R primers (see Table S2). PCR products representing three different mutation types, insertions, small deletions, and large deletions, were detected. We selected three insertion mutants, four small-deletion mutants, and three large-deletion mutants to confirm the position of the mutation by sequencing. The amplified 1,791-bp fragment from 10 mutants was cloned into the pBlueScript II SK(+) vector prior to sequencing to identify the mutation sites and complete sequencing.

### Plant inoculation.

In a greenhouse, rice plants (*Oryza sativa* cv. Milyang 23) were inoculated at the flowering stage with approximately 1 × 10^8^ CFU/ml of *B. glumae*. Disease symptoms in the rice plants were evaluated on day 7 after inoculation. The disease index was determined as described previously (41), using the following scale: 0 = healthy panicle, 1 = panicle 0 to 20% discolored, 2 = panicle 20 to 40% discolored, 3 = panicle 40 to 60% discolored, 4 = panicle 60 to 80% discolored, and 5 = panicle 80 to 100% discolored [disease index = Σ (number of samples per score × score)/the total number of panicles]. Pathogenicity assays were repeated three times with three replications.

### Electrophoretic mobility shift assay.

QsmR-His was purified as described previously (10). The upstream regions of putative QsmR target genes were amplified using the primers listed in Table S2. PCR products were labeled with biotin using a LightShift chemiluminescent electrophoretic mobility shift assay kit (Thermo Fisher Scientific), according to the manufacturer’s protocol. We used a 329-bp sequence upstream of *katE1* as competitor DNA. This DNA fragment was amplified using primers KatE1-F and KatE1-R (see Table S2). Purified QsmR-His (10 nM or 250 nM) was incubated in binding buffer [10 mM Tris-HCl (pH 7.5), 100 mM NaCl, 0.5 mM EDTA, 1 mM dithiothreitol [DTT], 5% (vol/vol) glycerol, and 10 ng µl^−1^ poly(dI-dC)] containing 1 nM or 2 nM biotin-labeled DNA for 15 min at 28°C. In competition analyses, unlabeled target DNA at 10-fold to 15-fold molar excess was added to each reaction mixture along with the extract before the addition of labeled DNA targets. Reaction mixtures were separated on nondenaturing 4% (wt/vol) gels and transferred to nitrocellulose membranes, followed by detection of relevant bands using streptavidin/horseradish peroxidase-derived chemiluminescence kits, as described by the manufacturer. Images were visualized using ChemiDoc XRS+ and Image Lab Software (Bio-Rad).

### Construction of *B. thailandensis aceA*::*lacZY* and *aceA*::*lacZY*/*aceA*.

The *aceA* (BTH_I1998) gene was deleted by integration of a suicide vector, as described previously (42). Briefly, a 250-bp DNA fragment encoding only the 5′ end of the *aceA* gene was generated by PCR using BTaceA-F and BTaceA-R primers, cloned into pBluescript II SK(+), and termed “pBTACE1.” A 250-bp DNA fragment obtained from pBTACE1 by digestion performed with EcoRI and XbaI was ligated into a suicide vector containing *lacZY* genes and pVIK112 to produce pBTACE2 (see Tables S1 and S2) (42). We mutagenized pBTACE2 by the use of EZ-Tn*5*(Tp) to replace kanamycin resistance with trimethoprim (Tp) resistance. pBTACE2_Tp was mobilized from DH5α (λ*pir*) into *B. thailandensis* E264 by conjugation using pRK2013 as a helper plasmid. The integrated mutant was confirmed by PCR using LacFuse and BTaceA-R primers (see Table S2). To complement the *aceA* mutant of *B. thailandensis*, we used the Tn*7* site-specific transposition machinery system (9) to insert a single copy of *aceA* downstream of *glmS*.

### Generation of S9NC5 and BICL39C.

For genetic complementation of *qsmR* mutant BGS9, we mutagenized the mutant via the use of mini-Tn*5rescue* (9) to integrate *qsmR* into the genome as a single copy. We confirmed that a single copy of the *qsmR* gene was inserted into the intergenic region between bglu_1g10240 and bglu_1g10250 in chromosome 1 of the *qsmR* mutant BGS9, resulting in strain S9NC5 (BGR1 *qsmR*::Ω/*qsmR*). Likewise, the *aceA* gene was inserted into the intergenic region between bglu_2g21870 and bglu_2g21880 in chromosome 2 of the *aceA* mutant BICL39, resulting in strain BICL39C (BGR1 *aceA*::EZ-Tn*5*/*aceA*).

### EZ-Tn*5* mutagenesis and marker exchange.

pICL1 and pPEP1 were mutagenized using a EZ-Tn*5* <DHFR-1> insertion kit (Epicentre, Madison, WI) and marker exchange, as described previously (10). All constructs were confirmed by Southern blot analysis. The EZ-Tn*5* insertion sites were determined by DNA sequencing according to the manufacturer’s protocols.

### Statistical analysis.

Statistical analyses were performed using SPSS version 22 (IBM Corp., Armonk, NY). Comparisons were performed by one-way analysis of variance (ANOVA), followed by Tukey’s multiple-comparison test (set at 5%). In Fig. S3A in the supplemental material, the data represented by the 2 bars were compared by unpaired *t* test.

### Accession number(s).

The NCBI GEO accession number for the RNAseq data series of BGR1, BICL39, and BICL39C is GSE70582.
