# Supplementary figures and images for: Lethal Consequences of Overcoming Metabolic Restrictions Imposed on a Cooperative Bacterial Population
Source: mBio. 2017 Feb 28;8(1):e00042-17. doi: 10.1128/mBio.00042-17 (PMC5347341; doi:10.1128/mBio.00042-17)

**A**

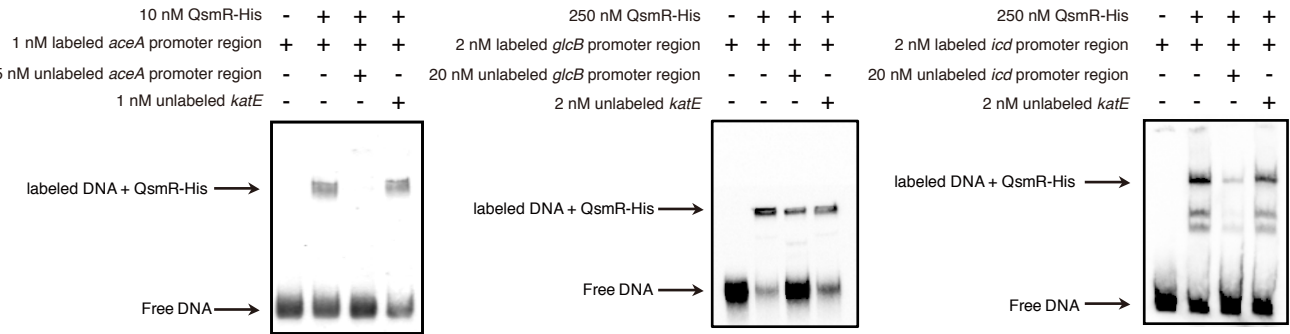

**B**

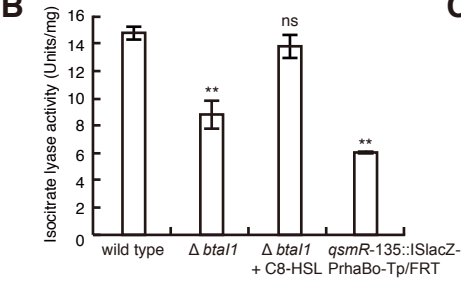

**C**

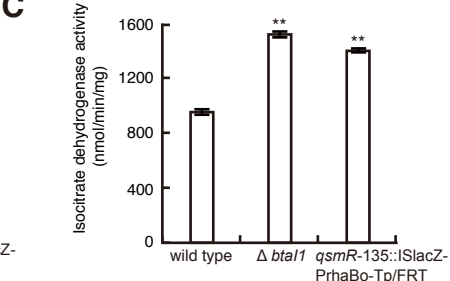

Supplement: FIG S1 [file mbo001173220sf1.pdf]

**A**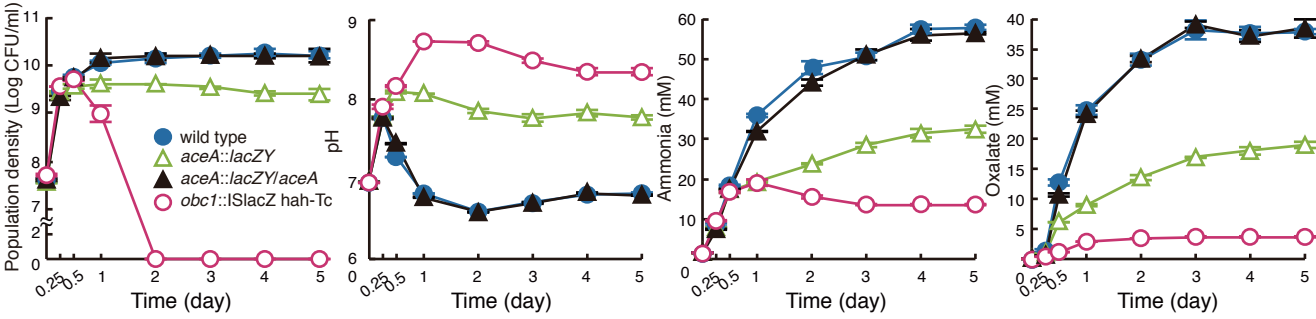**B**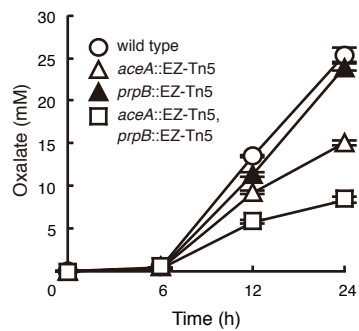

Supplement: FIG S2 [file mbo001173220sf2.pdf]

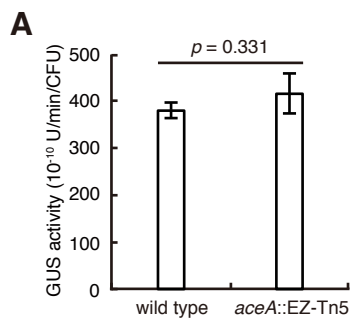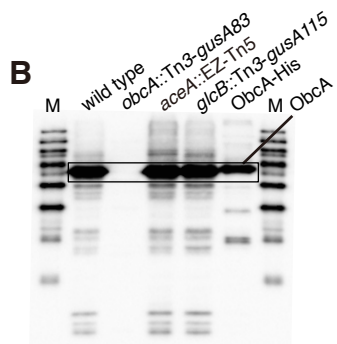

Supplement: FIG S3 [file mbo001173220sf3.pdf]

**A**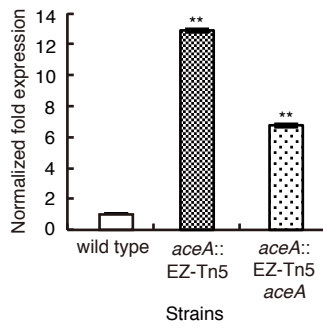**B**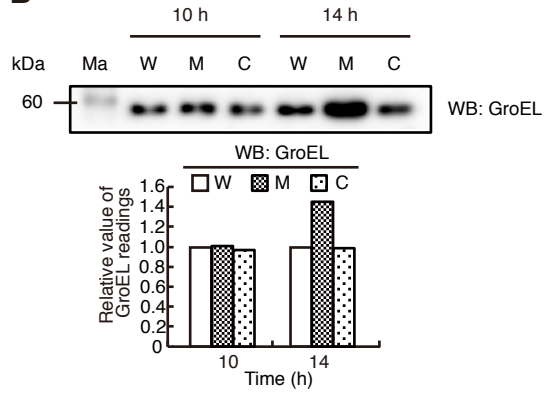

Supplement: FIG S4 [file mbo001173220sf4.pdf]

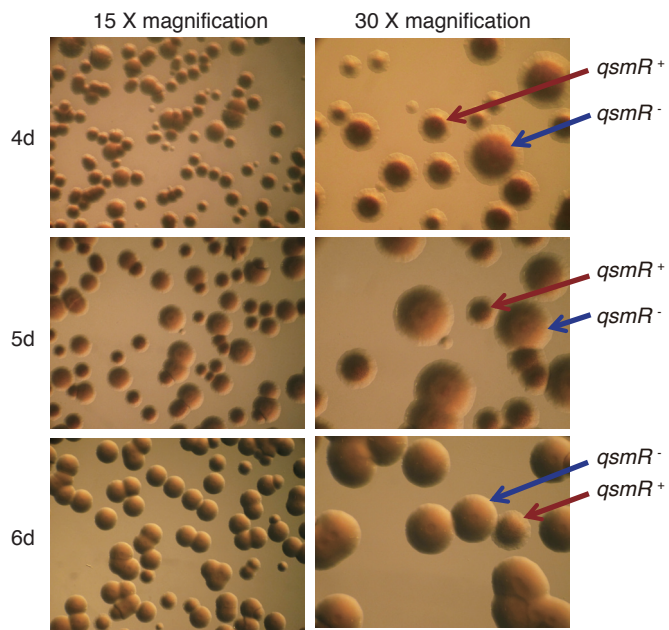

Supplement: FIG S5 [file mbo001173220sf5.pdf]

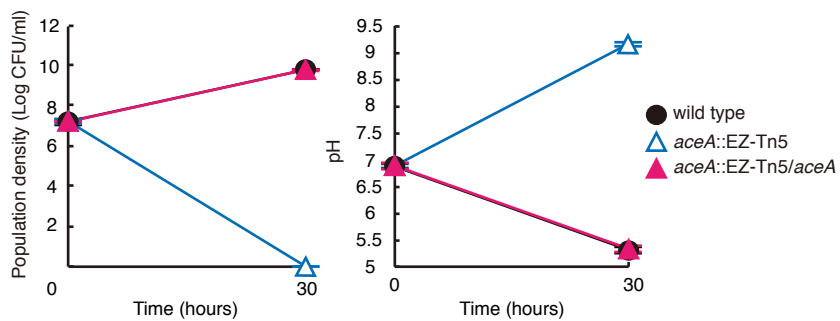

Supplement: FIG S6 [file mbo001173220sf6.pdf]

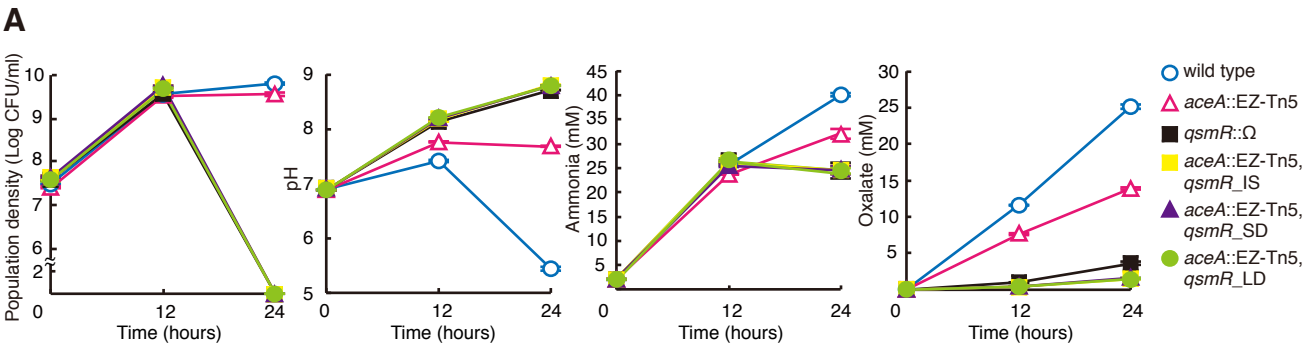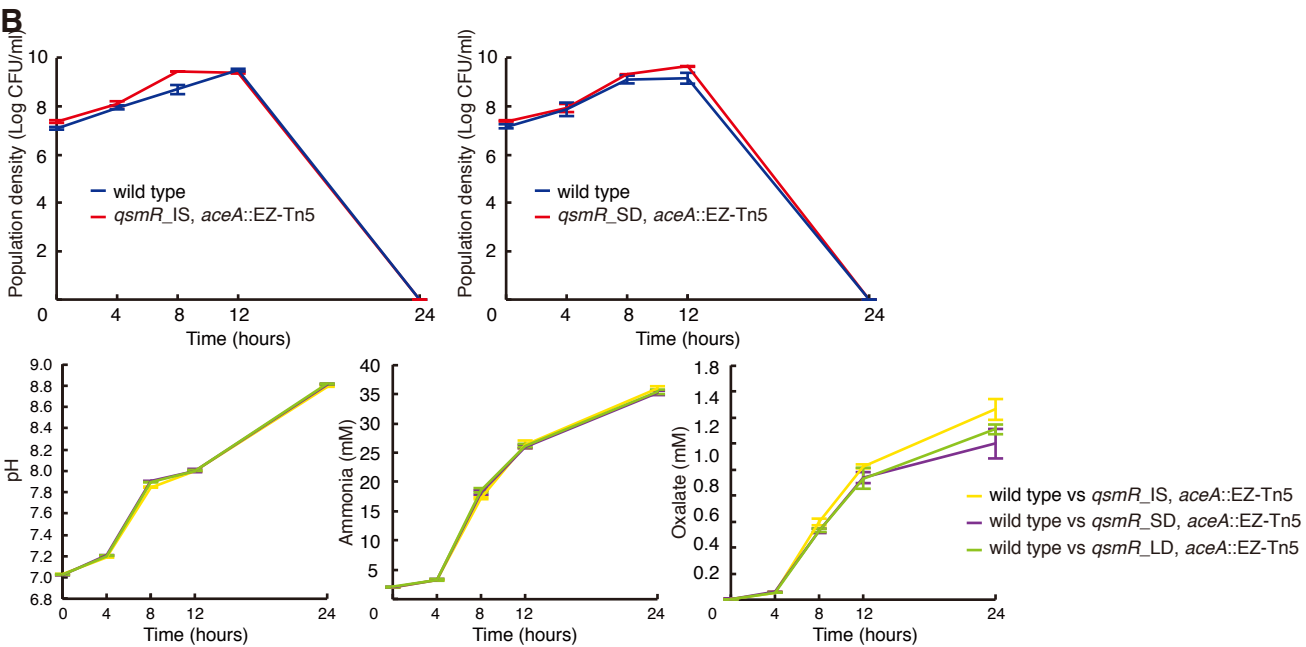

Supplement: FIG S7 [file mbo001173220sf7.pdf]

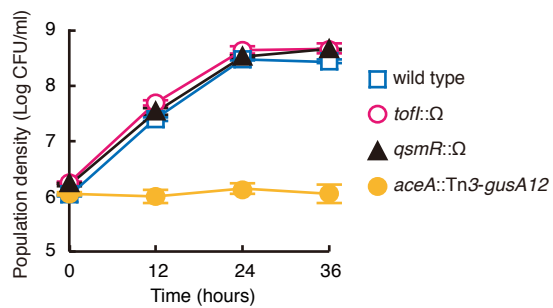

Supplement: FIG S8 [file mbo001173220sf8.pdf]
