# Supplementary material for: Lethal Consequences of Overcoming Metabolic Restrictions Imposed on a Cooperative Bacterial Population
Source: mBio. 2017 Feb 28;8(1):e00042-17. doi: 10.1128/mBio.00042-17 (PMC5347341; doi:10.1128/mBio.00042-17)
Supplement: TABLE S1 [file mbo001173220st1.docx]

**TABLE S1** Bacterial strains and plasmids used.

| *Strain or plasmid* | *Characteristics* | *Source or reference* |
| --- | --- | --- |
| *Burkholderia* *glumae* BGR1 | Wild type, Rif^R^ | (1) |
| BGS2 | BGR1 *tofI*::Ω | (1) |
| BGS9 | BGR1 *qsmR*::Ω | (2) |
| S9NC5 | BGR1 *qsmR*::Ω/*qsmR* | This study |
| BICL39 | BGR1 *aceA*::EZ-Tn*5* | This study |
| BICL39C | BGR1 *aceA*::EZ-Tn*5*/*aceA* | This study |
| BICD38 | BGR1 *icd1*::EZ-Tn*5* | This study |
| BACE31 | BGR1 *aceK*::Tn*3*-*gusA31* | This study |
| BOBA83 | BGR1 *obcA*::Tn*3*-*gusA83* | (3) |
| BMSG115 | BGR1 *glcB*::Tn*3*-*gusA115* | This study |
| BPRPB180 | BGR1 *prpB*::EZ-Tn*5* | This study |
| BICML | BGR1 *aceA*::Tn*3*-*gusA12, prpB*::EZ-Tn*5* | This study |
| BICOX | BGR1 *aceA*::EZ-Tn*5*, *obcA*::Tn*3*-*gusA83* | This study |
| BICL12 | BGR1 *aceA*::Tn*3*-*gusA12* | This study |
| BICL39_LY | Spontaneous mutant from batch cultures of *aceA*::EZ-Tn*5*, IS1418 insertion mutant | This study |
| BICL39_C | Spontaneous mutant from batch cultures of *aceA*::EZ-Tn*5*, IS1418 insertion mutant | This study |
| BICL39_IS | Spontaneous mutant from batch cultures of *aceA*::EZ-Tn*5*, IS1418 insertion mutant | This study |
| BICL39_SD | Spontaneous mutant from batch cultures of *aceA*::EZ-Tn*5*, small deletion in *qsmR* gene | This study |
| BICL39_LD | Spontaneous mutant from batch cultures of *aceA*::EZ-Tn*5*, large deletion in *qsmR* gene | This study |
| *B*. *thailandensis* E264 | Wild type | (4) |
| JBT101 | E264 Δ*btaI1* | (5) |
| BT09539 | E264 *qsmR*-135::ISlacZ-PrhaBo-Tp/FRT | (6) |
| BT00401 | E264 *obc1*-117::ISlacZ hah-Tc | (6) |
| BTICL | E264 *aceA*::*lacZY* | This study |
| BTICLC | E264 *aceA*::*lacZY/aceA* | This study |
| *Escherichia coli* DH5α | F^-^ Φ80d*lacZ*Δ*M15* Δ(*lacZYA-argF*)*U169 endA1 recA1 hsdR17* (r_K_^-^m_K_^+^) *deoR thi-1 supE44 λ-gyrA96 relA* | Gibco BRL, Waltham, MA, USA |
| S17-1 | Tra^+^, *recA*, Sp^R^ | (7) |
| DH5α (λpir) | λ*pir* enables oriR6K vector replication | (8) |
| CC118 (λpir) | Δ(*ara*-*leu*) *araD* _*lacX74 galE galK phoA thi-1 rpsE rpoB argE* (Am) *recA1*; lysogenized with λ*pir* phage | (9) |
| C2110 | *polA*, Nal^R^ | (10) |
| BL21(DE3) | F^-^*ompT hsdS_B_*(r_B_^-^m_B_^-^)*gal dcm* (DE3) | Novagen, Madison, WI, USA |
| Plasmids |  |  |
| pBluescript II SK(+) | Cloning vehicle; phagemid, pUC derivative, Amp^R^ | Stratagene, Santa Clara, CA, USA |
| pLAFR3 | Tra^-^, Mob^+^, RK2 replicon, Tet^R^ | (11) |
| pICL1 | 19.81 kb DNA fragment including the *aceA* gene from strain BGR1 cloned into pLAFR3 | This study |
| pIBXII | 3.3 kb *Xho*I fragment including *aceA* gene from pICL1 into pLAFR6, Tet^R^ | This study |
| pBTACE1 | 250 bp PCR fragment including the *aceA* gene from strain E264 cloned into pBluescript II SK(+) | This study |
| pBTACE2 | 250 bp *Eco*RI-*Xba*I DNA fragment from pBTACE1 clone into pVIK112 | This study |
| pIDH1 | 6.3 kb *Sac*I-*Hin*dIII DNA fragment including the *icd1* gene from strain BGR1 cloned into pBluescript II SK(+) | This study |
| pIDH3 | 6.3 kb *Sac*I-*Hin*dIII DNA fragment including the *icd1* gene from pIDH1 into pLAFR6 | This study |
| pACEK1 | 20.571 kb DNA fragment including the *aceK* gene from strain BGR1 cloned into pLAFR3 | This study |
| pPEP1 | 24.236 kb DNA fragment including the *prpB* gene from strain BGR1 cloned into pLAFR3 | This study |
| pMSG1 | 23.797 kb DNA fragment including the *glcB* gene from strain BGR1 cloned into pLAFR3 | This study |
| pRescue mini Tn5 | pUTKm oriV but without polylinker, enable rescue of the Tn*5* tagging region. | This study |
| pUC18R6K-mini-Tn7T-Tc | pUC derivate, replacement of ColE1 replicon with R6K replicon, Mini-Tn*7* delivery vector, Tet^R^ | (9) |
| pTNS2 | Helper plasmid for mini-Tn*7* transposition, Amp^R^ | (9) |
| pRK2013 | Helper plasmid, Tra ^+^, ColE1 replicon, Km ^R^ | (12) |
| pHoKmGus | Promoterless β-glucuronidase gene, Km^R^, Amp^R^ | (13) |
| pSShe | Cm^R^ | (10) |
| pET28b_obcA | *obcA* in pET28b, Km^R^ | (14) |
| pET28b_obc1*  pET28b_groEL | *obc1** in pET28b, Km^R^  *groEL* in pET28b, Km^R^ | (14)  This study |

**REFERENCES**

1. **Kim J, Kim JG, Kang Y, Jang JY, Jog GJ, Lim JY, Kim S, Suga H, Nagamatsu T, Hwang I.** 2004. Quorum sensing and the LysR-type transcriptional activator ToxR regulate toxoflavin biosynthesis and transport in *Burkholderia glumae*. Mol Microbiol **54**: 921–934.

2. **Kim J, Kang Y, Choi O, Jeong Y, Jeong JE, Lim JY, Kim M, Moon JS, Suga H, Hwang I.** 2007. Regulation of polar flagellum genes is mediated by quorum sensing and FlhDC in *Burkholderia glumae*. Mol Microbiol **64**: 165–179.

3. **Goo E, Majerczyk CD, An JH, Chandler JR, Seo YS, Ham H, Lim JY, Kim H, Lee B, Jang MS, Greenberg EP, Hwang I*.*** 2012. Bacterial quorum sensing, cooperativity, and anticipation of stationary-phase stress. Proc Natl Acad Sci USA **109**:19775–19780.

4. **Brett PJ, Deshazer D, Woods DE*.*** 1997. Chracterization of *Burkholderia pseudomallei* and *Burkholderia pseudomallei*-like strains. Epidemiol Infect **118**:137–148.

5. **Chandler JR, Duerkop BA, Hinz A, West TE, Herman JP, Churchill ME, Skerrett SJ, Greenberg EP.** 2009. Mutational analysis of *Burkholderia thailandensis* quorum sensing and self-aggregation. J Bacteriol **191**:5901–5909.

6. **Gallagher LA, Ramage E, Patrapuvich R, Weiss E, Brittnacher M, Manoil C*.*** 2013. Sequence-defined transposon mutant library of *Burkholderia thailandensis*. mBio **4**:e00604-13. doi: 10.1128/mBio.00604-13.

7. **Simon R, Priefer U, Pühler A*.*** 1983. A broad host range mobilization system for *in vivo* genetic engineering: transposon mutagenesis in Gram-negative bacteria. Nat Biotechnol **1**:784-791.

8. **Grant SG, Jessee J, Bloom FR, Hanahan D*.*** 1990. Differential plasmid rescue from transgenic mouse DNAs into *Escherichia coli* methylation-restriction mutants. Proc Natl Acad Sci USA **87**:4645-4649.

9. **Choi KH, Schweizer HP*.*** 2006. mini-Tn7 insertion in bacteria with secondary, non-*glmS*-linked *att*Tn7 sites: example *Proteus mirabilis* HI4320. Nat Protoc **1**:170-178.

10. **Stachel SE, An G, Flores C, Nester EW*.*** 1985. A Tn*3 lacZ* transposon for the random generation of β-galactosidase gene fusions: application to the analysis of gene expression in *Agrobacterium*. EMBO J **4**:891-898.

11. **Staskawicz B, Dahlbeck D, Keen N, Napoli C*.*** 1987. Molecular characterization of cloned avirulence genes from race 0 and race 1 of *Pseudomonas syringae* pv. *glycinea*. J Bacteriol **169**:5789-5794.

12. **Figurski DH, Helinski DR*.*** 1979. Replication of an origin-containing derivative of plasmid RK2 dependent on a plasmid function provided in *trans*. Proc Natl Acad Sci USA **76**:1648-1652.

13. **Bonas U, Stall RE, Staskawicz B.** 1989. Genetic and structural characterization of the avirulence gene avrBs3 from *Xanthomonas campestris* pv. *vesicatoria*. Mol Gen Genet **218**: 127–136.

14. **Oh J, Goo E, Hwang I, Rhee S.** 2014. Structural basis for bacterial quorum sensing-mediated oxalogenesis. J Biol Chem **289**:11465–11475.
