# Supplementary material for: Lethal Consequences of Overcoming Metabolic Restrictions Imposed on a Cooperative Bacterial Population
Source: mBio. 2017 Feb 28;8(1):e00042-17. doi: 10.1128/mBio.00042-17 (PMC5347341; doi:10.1128/mBio.00042-17)
Supplement: TABLE S2 [file mbo001173220st2.docx]

**TABLE S2** Primers used in this study.

| *Primer* | *Sequence* |
| --- | --- |
| BTaceA-F | TCAACAACAGGCTCAGGAAC |
| BTaceA-R | ACAGATAGATCGCCTTGAGG |
| LacFuse | GGGGATGTGCTGCAAGGCG |
| glmS-down | AGCCGCAGATCATCGCCTG |
| 1g32800-up | CCACGCATCGAAATCCTC |
| P_Tn7R_ | CACAGCATAACTGGACTGATTTC |
| aceA-F | GGACCAGTCGCTTTATCCGGC |
| aceA-R | GAAGTAGTCCACGTAGCCTT |
| glcB-F | CAGCGTCACGGGCTCAAGATAG |
| glcB-R | CATGGACGATCGCGGAGAAAC |
| iclF | GCGGGCGATCTCACGAC |
| iclR | GATGAGCTCCTTGATGGATTG |
| mgs_pair2_F | GTCGGCGTATCGGGCATGAA |
| mgs_pair2_R | CGTGACGCTGGATCATGGAAT |
| picd1-F | GTGCCGAACGGTTTGTTGA |
| picd1-R | CATGGTCGACTCCAGTGATATG |
| KatE1-F | ACTCGCGCCGCTCGTCGAA |
| KatE1-R | ACATCGGCATCCTGGGTCGC |
| qsmR-F | GAAATGAGGGAGACCAGTCTGTCTATT |
| qsmR-R  groEL-F  groEL-R  NdeI_groEL  groEL_HindIII | GTTTGCGGTTCCGGGTTATTCATGTTCGATCF  ATGGCAGCTAAAGACGTC  ACGAACGCTCGAGCACCACGTT  CGCATATGGCAGCTAAAGACGTCGTGTTC  GGAAGCTTCATGTCCATGCCCATGCCGCCCAT |
